# Comparative efficacy of palliative radiotherapy dose schedules in advanced bladder cancer-associated gross hematuria

**DOI:** 10.3332/ecancer.2024.1718

**Published:** 2024-06-21

**Authors:** Kashif Ali Sarwar, Sameed Hussain, Ahsan Mahmood, Zeeshan Ahmed Alvi, Ateeqa Saad

**Affiliations:** Department of Radiation Oncology, Combined Military Hospital, National University of Medical Sciences, The Mall, Rawalpindi 46000, Pakistan; a https://orcid.org/0000-0001-7746-0724

**Keywords:** palliative radiotherapy, bladder cancer, hematuria, efficacy, hypofractionated, single fraction

## Abstract

**Introduction:**

Gross hematuria (GH) in advanced/inoperable bladder cancer patients causes significant morbidity. Patients frequently need multiple transfusions. Hypofractionated radiotherapy (RT) has been shown to be effective in symptom palliation. In this study, we explore the efficacy of various fractionation regimens in these patients.

**Methods:**

This single institute retrospective analysis was conducted on 60 consecutive patients treated with palliative RT. Fractionation (single versus multiple) and biologically equivalent doses (BED; high ≥36 Gy versus low <36 Gy) were used to compare the efficacy of various fractionation regimens. The primary outcome was the difference in objective response rate (ORR) between various strata at 2, 4, 8 and 12 weeks. Major secondary outcomes were differences in ORR according to Eastern Cooperative Oncology Group (ECOG) performance status (PS) and tumour node metastases (TNM) stage, and the proportion of patients requiring re-transfusion(s) at 12 weeks. Data were analysed using SPSS 23.

**Results:**

Overall ORR at 2, 4, 8 and 12 weeks was 86%, 77%, 67% and 55%, respectively. There was no statistically significant difference in response rates between single or multi-fraction, or high versus low BED groups (All *p* = >0.05). Moreover, ECOG PS (*p* = 0.11) or TNM stage (*p* = 0.58) also had no impact on the response rate at 12 weeks. Nearly one-third (31%) of patients required further transfusions at 12 weeks.

**Conclusion:**

RT is an effective modality to control GH. No difference in ORR was found between single fractions versus multiple fractions, or high versus low BED regimens. Single fraction RT can be offered to these patients considering low cost, patient convenience and minimal side effects.

## Introduction

Bladder cancer is the tenth most common malignancy overall and sixth most common in men, constituting about 4.4% of all tumours making it a major cause of morbidity and mortality worldwide [1]. Painless or painful gross hematuria (GH) is the most common presenting complaint and is often neglected, often treated initially as lower urinary tract infection or calculi, particularly in our part of the world where health access is a challenge for many. While radical cystectomy or chemoradiotherapy are the main treatment options in curative settings and good performance status (PS) patients, palliative management is often the treatment of choice in advanced or unresectable tumours and those patients with poor PS. Although there are many treatment options for these patients, including chemotherapy, immunotherapy and/or radiation, most patients progress symptomatically during treatment. Unrelenting GH not only results in frequent hospital visits but also impacts the quality of life (QOL) of patients, causing major morbidity. Patients often require multiple transfusions. A variety of local treatment options can be employed, including endovascular radio or chemo-embolization, radiofrequency ablation, cryoablation, argon laser coagulation, intravesical fulguration and radiotherapy (RT) [2–4]. RT is often employed later in treatment, as a minimally invasive treatment option, to control hematuria that is not responsive to other local measures. Its use has been employed with variable success in other sites as well, including stomach and lung, to control intractable hematemesis and hemoptysis, respectively [5]. RT to palliate bleeding can be effective within 24–48 hours of delivery of the first dose, making it a safe and appropriate tool for rapid symptom control. Contemporary studies have shown response rates ranging from 39% to 95% in palliation of GH in bladder cancer patients using various RT regimens [6–9]. Although a variety of RT dose schedules are commonly used, none is standard. Studies have shown variable results in favour of high (>36 Gy) biologically effective dose (BED) RT schedules [10] but a recent meta-analysis showed that no schedule is superior to others in terms of response rates [11]. However, the same meta-analysis and a few other studies mentioned earlier [6, 10] indicate that longer and/or high BED schedules are associated with greater hematuria recurrence-free intervals and greater freedom from transfusions, albeit at the cost of more dysuria. While longer schedules can potentially be favoured in patients to reduce dependency on subsequent transfusion(s), low BED schedules offer more convenience to the patients, are cost-effective and carry less risk of worsening lower urinary symptoms. We retrospectively try to explore the differences, if any, in a cohort of patients treated and followed up at our department.

## Patients and methods

This is a retrospective analysis conducted at the Department of Radiation Oncology, Combined Military Hospital, National University of Medical Sciences, Rawalpindi.

### Eligibility and inclusion criteria

All consecutive, inoperable, locally advanced/advanced bladder cancer patients, who received palliative RT to control GH, between January 2020 and September 2023, were included and evaluated. Patients aged 18 years and above who had a histopathological diagnosis of urothelial bladder cancer and were being treated with palliative intent either due to advanced disease or poor PS, were eligible. Patients must have GH as their primary symptom (grade 2 or more per CTCAE v1.1). Those who had received chemotherapy or immunotherapy previously were eligible. Patients who received previous non-pelvic or pelvic RT for any reason(s) other than GH were also eligible. Variant histologies (defined as >95% component being composed of non-urothelial histology, e.g., Pure squamous or pure adenocarcinoma), and those for whom bladder RT was given for local control instead of palliation of hematuria, were excluded. Staging is according to the 8th edition of the AJCC/UICC tumour node metastases (TNM) tumour staging manual.

### Treatment and data collection

All patients underwent a planning computed tomography (CT) scan in supine treatment position with knee and ankle support. Scans were done in the empty bladder and rectum. The ECLIPSE 16.1 treatment planning software (Varian Inc. Palo Alto, CA, USA) was used for contouring and treatment planning. The entire bladder was contoured as the clinical target volume (CTV). An isotropic margin of 1–2 cm was given around the CTV to generate the planning target volume. 3D conformal RT was utilised. All treatments were delivered using 6–10 MV photons from a Varian CLINAC DHX 2300. Multileaf collimators were utilised for beam shaping. The radiation fractionation regimen used was at the discretion of the treating physician. Five types of dose fractionation regimens were used; 36 Gy in 6 weekly fractions (BED_10_ 57.6), 30 Gy in 10 fractions (BED_10_ 39), 21 Gy in 3 fractions on alternate days (BED_10_ 35.7), 20 Gy in 5 fractions (BED_10_ 28) and 8 Gy in single fraction (BED_10_ 14.4).

Data were obtained from patients’ electronic medical records stored in the Accessible Rich Internet Applications (ARIA) oncology information system developed by Varian Inc., California, USA. Patient-related variables included age, gender, Eastern Cooperative Oncology Group (ECOG) PS, tumour histology and stage (locally advanced versus metastatic). Treatment-related variables collected included dose fractionation (Single fraction versus multiple fractions), and biological effective dose BED (high BED >36 Gy versus low BED < 36 Gy).

### Outcomes

The primary outcome variable is the overall objective response rate (ORR) for GH after RT, as well as an assessment of ORR in high BED versus low BED and single versus multiple fraction regimens. The response was defined as either complete, i.e., no GH and no further requirement of erythrocyte transfusion(s) 2 weeks after completion of RT, or partial, i.e., a decrease in the frequency of hematuria and no requirement of erythrocyte transfusion(s) 2 weeks after completion of RT. Patients whose hematuria remained the same or worsened and/or whose need for transfusions increased, or lost to follow-up due to any cause, were categorised in the no response category. The responses were evaluated 2, 4, 8 and 12 weeks after completion of RT.

The main secondary outcome was the proportion of patients who were hematuria free at 12 weeks in both high versus low BED and single versus multiple fraction cohorts. Other secondary outcomes were differences (if any) in ORR according to ECOG PS and TNM stage, the proportion of patients requiring re-transfusion(s) at 12 weeks, and the most common time period to relapse after response.

### Statistical analysis

For the primary outcome variable, the proportion of patients who responded to treatment has been reported. The difference in ORR between the two groups (high versus low BED, Single fraction versus multiple fractionation schemes) was determined by Fisher exact test/Pearson’s chi-square where criteria were fulfilled. The difference in ORR according to ECOG PS and TNM stage was analysed using the same tests. Other secondary outcomes are reported descriptively. SPSS version 23 was used to analyse data.

The study was approved by the Institutional ethical review committee. Since it is a retrospective study, it waived the need for informed consent from individual patients. No identifying information has been revealed in the present study.

## Results

From January 2020 till September 2023, a record of a total of 60 advanced/inoperable bladder cancer patients having GH as their chief complaint were treated at our hospital. The medical records of these patients were reviewed retrospectively to evaluate their data. Median age was 71 and all except 4 patients were male. The vast majority (87%) of patients had ECOG PS 2 or more. 90% of patients had urothelial histology and 75% of patients had metastatic disease. For the purpose of the study, treatments were grouped into two categories; high BED ≥36 Gy versus low BED <36 Gy, and single fraction versus multi fraction. 70% of patients received low BED while the rest underwent high BED RT. Similarly, 37% of patients received single-fraction RT and 63% multi-fraction RT. Patient demographic and treatment-related data is summarised in Table 1.

**Figure 1.**
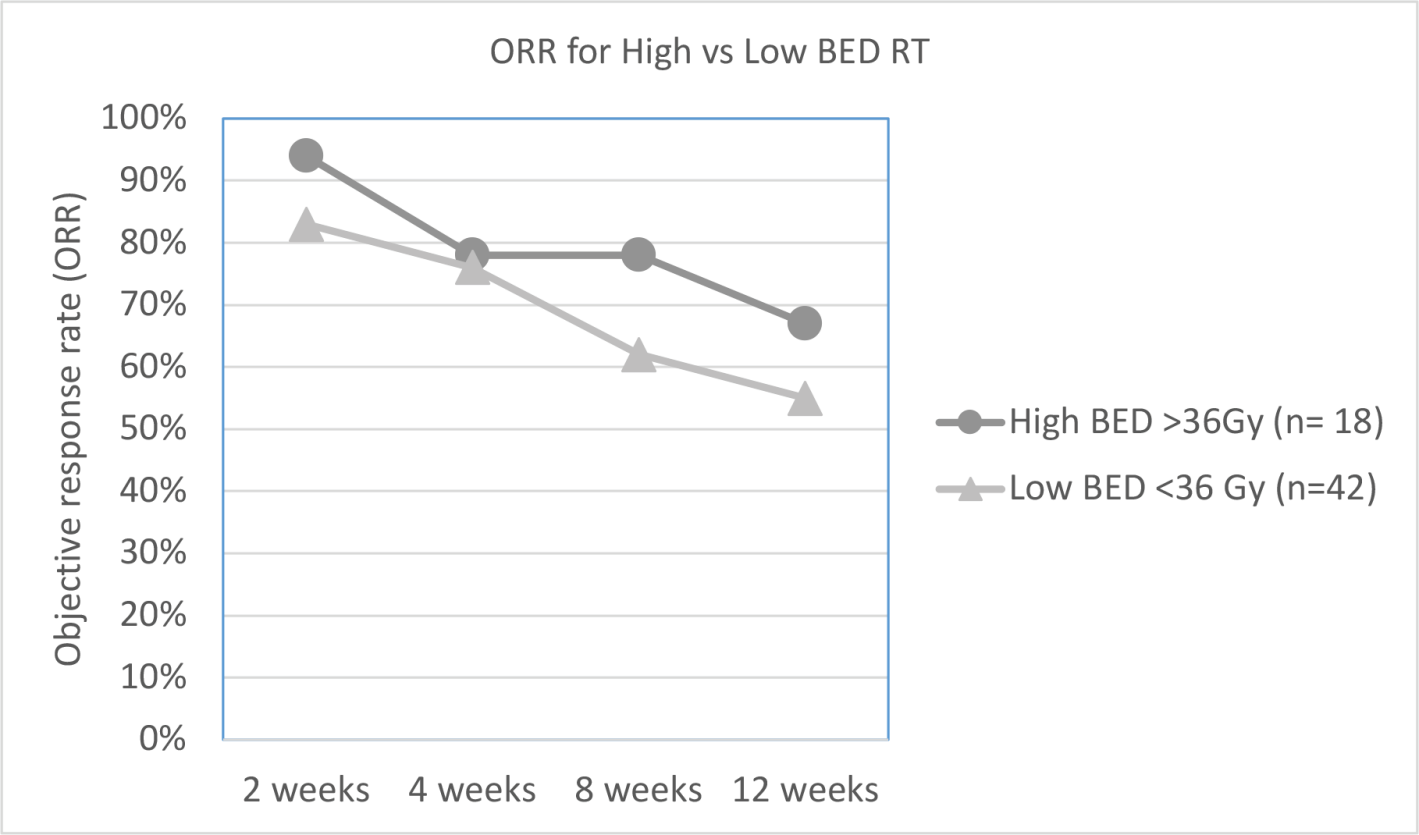
Line graph showing trend of ORR for high versus low BED regimens.

**Figure 2.**
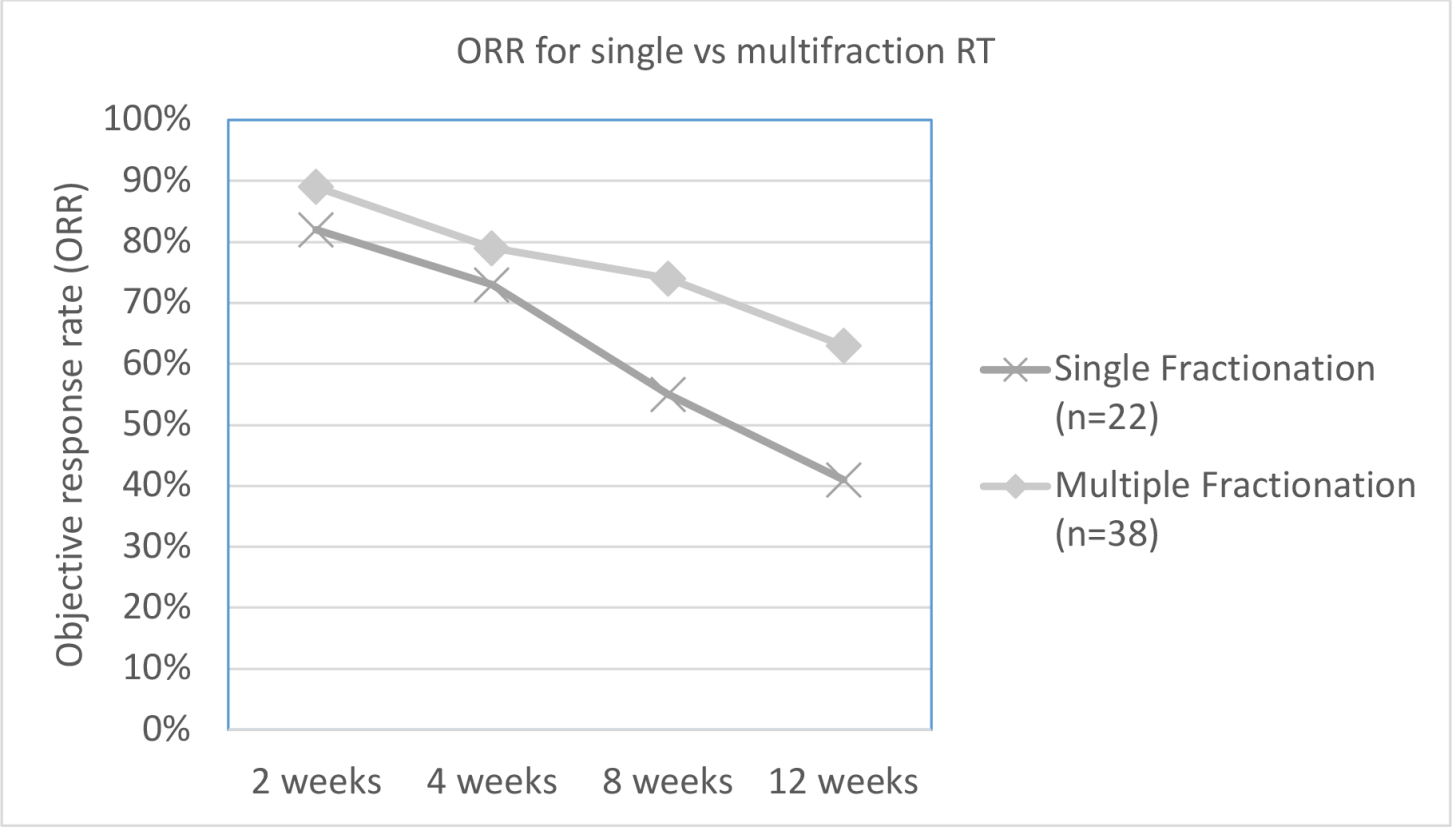
Line graph depicting difference in ORR between single versus multi-fraction regimens over observed time.

As per the definition of response described in the methods section, overall ORR at 2, 4, 8 and 12 weeks was 86%, 77%, 67% and 55%, respectively. The difference in ORR at various time intervals between low versus high BED, and single versus multi-fraction regimens are explained in Table 2, and shown in Figures 1 and 2. As it can be appreciated, there is no statistically significant difference in response rates between these two groups at any time interval, though there is a trend toward more sustained response for high BED/multi-fraction schedules.

**Figure 3.**
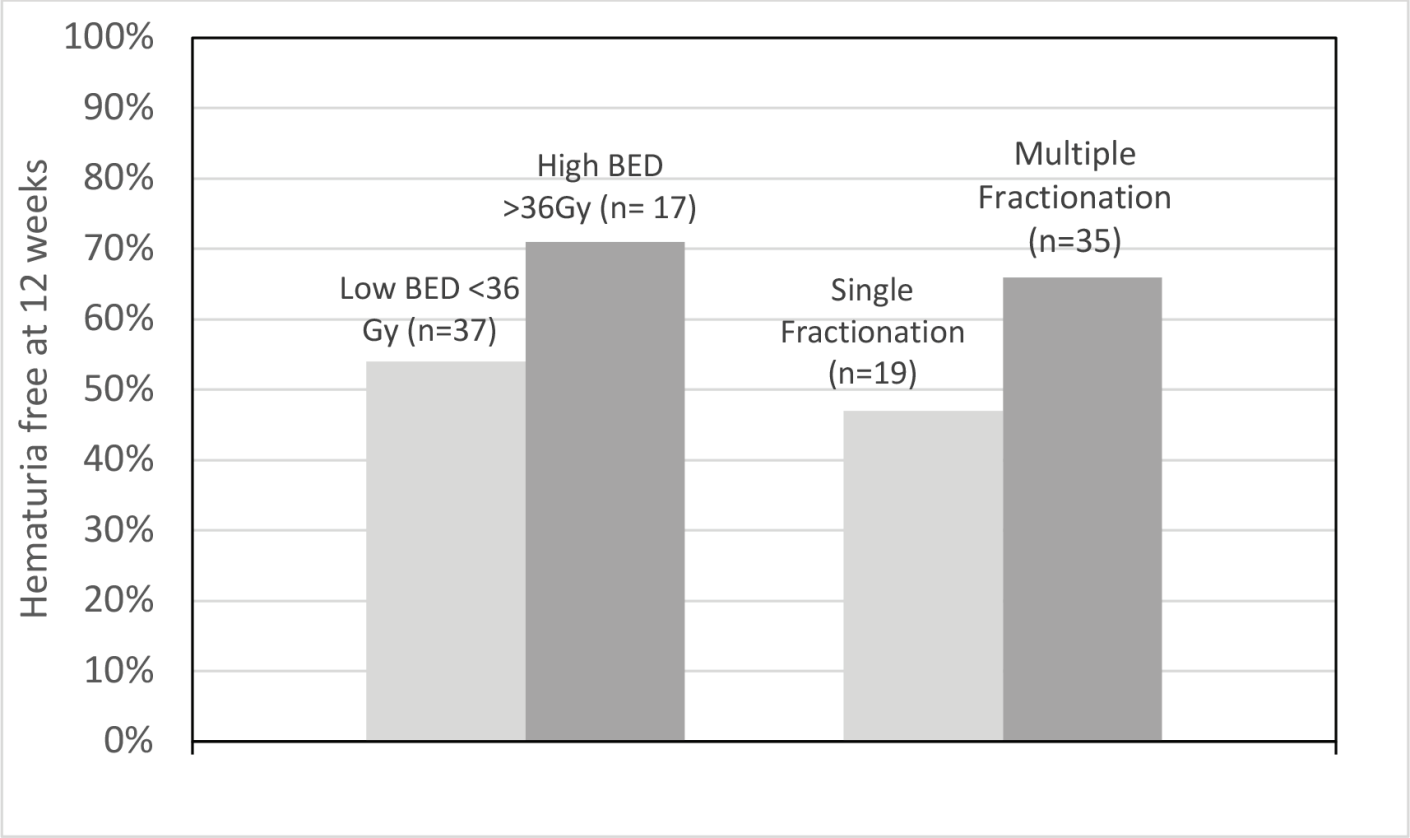
Cluster graph showing proportion of patients who were hematuria-free at 12 weeks.

In the 54 patients whose data were evaluable at 12 weeks for recurrence, GH recurred/worsened within 12 weeks in 46% of patients in the low BED group, while the same was 29% in the high BED cohort (*p* value = 0.25). Similarly, in single versus multi-fraction cohorts, these values were 53% and 34% (*p* value = 0.19), respectively. This is shown in Figure 3.

Overall, 17 (31%) patients required further transfusions at 12 weeks’ assessment while the rest needed no erythrocyte transfusions till 3 months’ post RT. During this time, 14 (26%) patients relapsed after at least some response. The relapse time was stratified into 3 groups, i.e., 2–4, 4–8 and 8–12 weeks. 43% (6) of relapses were during 4–8 weeks after RT completion, while other time intervals had 28% (4) each. This shows that the most common period to relapse was between 4 and 8 weeks after completion of RT. There was no statistically significant different in time to relapse between low BED versus high BED (*p* value = 0.19), or single versus multi-fraction regimens (*p* value = 0.45).

No difference was found between response rates at 12 weeks if data were stratified for ECOG PS (*p* value = 0.11) or TNM (*p* value = 0.58).

## Discussion

RT is utilised in many sites for symptom palliation; its utility is heterogeneous, ranging from pain control and prevention of skeletal-related events to bleeding palliation. This retrospective study shows that palliative hypo-fractionated RT can provide effective bleeding control in advanced inoperable bladder cancer, and can improve patient’s QOL by decreasing dependence on repeated transfusions. Overall response rates up to 86% and 77% at 2 and 4 weeks were achieved, respectively, with a reduction in the requirement for transfusions. This fulfills the primary objective of rapid symptom palliation from the patients’ perspective. Although it appeared that responses were more durable with longer courses, it was statistically not significant in our analysis, possibly due to a small sample size or shorter duration of available follow-up records.

Medical Research Council BA-09 trial is the only randomised controlled trial (RCT) to date that specifically randomised patients to two treatment regimens (35 Gy in 10 fractions versus 21 Gy in 3 fractions), but it was primarily a trial for local disease control in medically inoperable patients, who generally have a better prognosis and symptom load than those having advanced disease and uncontrolled/recurrent GH [12]. Nevertheless, this was the first robust evidence that hypo-fractionated regimen is as effective as long schedules. The meta-analysis by Tey *et al* [11] provides the most comprehensive data on optimal dose fractionation and indicates that higher doses are not associated with improved response rates. However, longer schedules led to more durable hematuria control rates which might be beneficial for patients with better PS and longer life expectancy [11].

While certain studies showed that longer courses/high BED RT regimens provide more durable symptom control [6, 10], others indicate the fact that both types of regimen have high recurrence rates, ranging from 41% to 69% at a median of 4–6 months [7, 8]. Moreover, hypo-fractionated schedules are cost-effective, convenient and carry a lower risk of treatment-related adverse effects. Though it is difficult to run RCTs in these scenarios, owing to selection bias and ethical issues among others, in a few other sites like in bone metastases, shorter and even single fraction radiation courses have been advocated in a randomised setting [13–15]. All these studies encourage utilising shorter courses of RT, particularly in patients with limited life expectancy for whom symptom control is the priority rather than long-term local control. Interestingly, no study has specifically explored the utility and efficacy of single fraction RT for GH palliation. Only two retrospective studies included patients treated with 8 Gy in a single fraction [6, 16]. In both studies, there was no statistically significant difference between the two regimens for hematuria control on multivariate analysis. However, there was no stratification for single versus multi-fraction RT. Our study indicates that it is as efficacious as multi-fraction regimens, with a potential risk of more recurrences. Needless to say, RT is a safe and effective mode of symptom palliation in advanced bladder cancer, yet it is under-utilised. A survey conducted in Japan by the Japanese Radiation Oncology Study Group highlighted the under-utilisation of RT by radiation oncologists for GH and emphasized the need to employ it as a safe, fast and effective palliation modality [17]. The aforementioned study also highlights a wide spectrum of dose fractionation techniques employed by physicians.

Our study had certain limitations. First, it was a retrospective and not a randomised controlled study. Second, consecutive treated patients were analysed. The choice of fractionation was up to the treatment physician considering both patient and disease-related factors, which might have introduced selection bias. Nevertheless, to the best of the authors’ knowledge, our study had the highest number of patients treated with a single fraction RT amongst all the studies published to date and the acute response rate appears to be similar to protracted courses. It provides the real world data of physician selected patients. Single-fraction RT for symptom control has not been explored sufficiently in bladder cancer-associated hematuria. This is of particular significance as many patients have poor PS and/or very little life expectancy (median approximately 3–5 months [6, 16]); hence, long-term control is not the primary goal. Effective palliation with minimum patient inconvenience should be the intent of treatment which a single fraction appears to fulfill. Cost-effectiveness is an added advantage, especially in resource-constrained countries like ours. Moreover, a single fraction can also be repeated if such a need arises. Future studies might explore this option and more robust data are needed, like in bone metastases, where there has been a worldwide tilt toward shorter RT courses and wider acceptance among the oncology community.

## Conclusion

Single fraction RT for control of GH in advanced bladder cancer is a viable treatment modality that not only provides effective symptom palliation but is cost-effective and convenient too. Since symptom palliation with minimum patient inconvenience is usually the intent of treatment in such patients, single fraction RT can be offered to these patients, especially in resource-constrained setups.

## List of abbreviations

AJCC, American Joint Committee on Cancer Control; CTCAE, Common terminology criteria for adverse events; CTV, Clinical target volume; ECOG, Eastern Cooperative Oncology Group; Gy, Gray; PS, Performance status; PTV, Planning target volume; RCT, Randomized controlled trial; TNM, Tumor node metastases; UICC, Union for International Cancer Control.

## Conflicts of interest

The authors declare none.

## Funding

This research received no specific grant from any funding agency, commercial or not-for-profit sectors.

## Ethics statement

The authors assert that all procedures contributing to this work comply with the ethical standards of the relevant national guidelines on human experimentation and with the Helsinki Declaration of 1975, as revised in 2008, and has been approved by the institutional ethical review board (CMH Rawalpindi IRB approval no 498/23). No violation of patient safety or privacy, breach of data, or human/institutional rights has been done.

## Author contributions

Kashif Ali Sarwar: Conception and design, data collection, data interpretation and analysis, drafting, final approval.

Ahsan Mahmood: Conception and design, data collection, drafting, peer review, final approval.

Sameed Hussain: Conception and design, data interpretation and analysis, drafting, peer review, final approval.

Zeeshan Ahmad Alvi: Conception and design, data interpretation and analysis, drafting, peer review, final approval.

Ateeqa Saad: Conception and design, data collection, drafting, peer review, final approval.

All authors agree to be accountable for all aspects of this study.

## Figures and Tables

**Table 1.** Summary of patient demographic and treatment-related data.

Demographic and treatment descriptors			
Age (years) Range Median	32–88 71	Histology Urothelial Mixed	54 (90%) 6 (10%)
Gender (*n*) Male Female	56 04	Previous RT Yes No	7 (12%) 53 (88%)
ECOG PS 0 or 1 2 3 4	8 (13%) 23 (38%) 22 (37%) 7 (12%)	Previous/current chemotherapy/Immunotherapy Yes No	26 (43%) 34 (57%)
Stage Locally advanced Metastatic	15 (25%) 45 (75%)	Mean pre RT hemoglobin	7.8 g/dL
RT Regimens (*n*) 8 Gy in single Fx Others: 20 Gy in 5 Fx OD 21 Gy in 3 Fx QOD 30 Gy in 10 Fx OD 36 Gy in 6 Fx q1w	22 (37%) 9 (15%) 11 (18%) 10 (17%) 8 (13%)	Mean post RT hemoglobin (2 weeks)	8.8 g/dL
BED High (≥36) Low (<36)	18 (30%) 42 (70%)		

**Table 2.** ORR at 2, 4, 8 and 12 weeks for various dose regimens and fractionation protocols.

BED	Response evaluation (%)			
	2 weeks	4 weeks	8 weeks	12 weeks
High >36 Gy (*n* = 18)	94%	78%	78%	67%
Low <36 Gy (*n* = 42)	83%	76%	62%	55%
Statistical significance	*p* = 0.41	*p* = 1.00	*p* = 0.37	*p* = 0.23
Fractionation				
Single (*n* = 22)	82%	73%	55%	41%
Multiple (*n* = 38)	89%	79%	74%	63%
Statistical significance	*p* = 0.45	*p* = 0.58	*p* = 0.13	*p* = 0.09

(*p* = *p* values)

## References

[1] Sung H, Ferlay J, Siegel RL (2021). Global cancer statistics 2020: GLOBOCAN estimates of incidence and mortality worldwide for 36 cancers in 185 countries. CA Cancer J Clin.

[2] Loffroy R, Pottecher P, Cherblanc V (2014). Current role of transcatheter arterial embolization for bladder and prostate hemorrhage. Diagn Interv Imaging.

[3] Sun L, Zhang W, Liu H (2014). Computed tomography imaging-guided percutaneous argon-helium cryoablation of muscle-invasive bladder cancer: initial experience in 32 patients. Cryobiology.

[4] Ghahestani SM, Shakhssalim N (2009). Palliative treatment of intractable hematuria in context of advanced bladder cancer. Urol J.

[5] Johnstone C, Rich SE (2018). Bleeding in cancer patients and its treatment: a review. Ann Palliat Med.

[6] Tey J, Soon YY, Cheo T (2019). Efficacy of palliative bladder radiotherapy for hematuria in advanced bladder cancer using contemporary radiotherapy techniques. In Vivo.

[7] Lacarrière E, Smaali C, Benyoucef A (2013). The efficacy of hemostatic radiotherapy for bladder cancer-related hematuria in patients unfit for surgery. Int Braz J Urol.

[8] Zhang H, Hojo H, Parshuram Raturi V (2020). Palliative radiation therapy for macroscopic hematuria caused by urothelial cancer. Palliat Med Rep.

[9] Kouloulias V, Tolia M, Kolliarakis N (2013). Evaluation of acute toxicity and symptoms palliation in a hypofractionated weekly schedule of external radiotherapy for elderly patients with muscular invasive bladder cancer. Int Braz J Urol.

[10] Ogita M, Kawamori J, Yamashita H (2021). Palliative radiotherapy for gross hematuria in patients with advanced cancer. Sci Rep.

[11] Tey J, Ho F, Koh WY (2021). Palliative radiotherapy for bladder cancer: a systematic review and meta-analysis. Acta Oncol (Madr).

[12] Duchesne GM, Bolger JJ, Griffiths GO (2000). A randomized trial of hypofractionated schedules of palliative radiotherapy in the management of bladder carcinoma: results of medical research council trial BA09. Int J Radiat Oncol Biol Phys.

[13] Harstell WF, Scott CB, Bruner DW (2005). Randomized trial of short- versus long-course radiotherapy for palliation of painful bone metastases. J Natl Cancer Inst.

[14] van den Hout WB, Linden YM, Steenland E (2003). Single- versus multiple-fraction radiotherapy in patients with painful bone metastases: cost-utility analysis based on a randomized trial. J Natl Cancer Inst.

[15] Hoskin P, Misra V, Hopkins K (2017). SCORAD III: randomized noninferiority phase III trial of single-dose radiotherapy (RT) compared to multifraction RT in patients (pts) with metastatic spinal canal compression (SCC). J Clin Oncol.

[16] Ali A, Song YP, Mehta S (2019). Palliative radiation therapy in bladder cancer – importance of patient selection: a retrospective multicenter study. Int J Radiat Oncol Biol Phys.

[17] Kosugi T, Shikama N, Saito T (2016). A nationwide survey in Japan of palliative radiotherapy for bleeding in gastrointestinal and genitourinary tumor patients. World J Oncol.

